# Complications and their management in the surgical treatment of Lipohyperplasia dolorosa. English version

**DOI:** 10.1007/s00105-022-05075-5

**Published:** 2022-12-22

**Authors:** Manuel Cornely, Matthias Gensior

**Affiliations:** 1 CG Lympha, Specialist Clinic for Operative Lymphology, Cologne, Germany; 2 LY. SEARCH, Biberstr. 7, 50678 Cologne, Germany

**Keywords:** Lymphological Liposculpture, Liposuction, Lipedema, Seroma, Erysipelas, Lymphologische Liposculptur, Liposuction, Lipödem, Serom, Erysipel

## Abstract

**Background:**

There are both conservative and surgical treatment options for Lipohyperplasia dolorosa (LiDo). A procedure that has been established since 1997 is the surgical treatment through Lymphological Liposculpture according to Cornely™.

**Aim:**

After extensive suctioning of the extremities, an extensive subcutaneous wound cavity with a trabecular connective tissue scaffold remains. Nevertheless, surgery-related complications are rare. Postoperative management and administration of antibiotics and antithrombotics are reviewed. The therapies for complications are presented in detail.

**Materials and methods:**

Retrospectively, the frequencies of adverse events in 1400 LiDo surgeries in 2020 were evaluated. The mean age of the patients was 47.81 years (range 16–78 years). Symmetrically, 504 outer legs (outer half of the limb [BO]), 504 inner legs (inner half of the limb [BI]), and 392 arms [A] were surgically treated.

**Results:**

Relevant adverse events rarely occurred: infections (1.79%), seromas (0.79%), erysipelas (0.28%), necrosis (0.14%) and deep vein thrombosis (0.07).

**Discussion:**

We were able to reduce the rate of postoperative complications to 3.07% in the Lymphological Liposculpture™ regime for the surgical treatment of LiDo. In their meta-analysis on liposuction, Kanapathy et al. reported an overall incidence of major surgical complications of 3.35%. The overall incidence of minor surgical complications was 11.62%, with seroma (5.51%) being the most common minor complication [26]. Kruppa et al. report that the liposuction procedure including fat removal for esthetic reasons has a complication rate of 9.5%. Wound infections with 4.5% and the formation of erysipelas with 4% are clearly in the foreground [20].

## Abstract (de)

**Hintergrund:**

Für die Lipohyperplasia dolorosa (LiDo) gibt es sowohl konservative als auch operative Behandlungsmöglichkeiten. Ein seit 1997 etabliertes Verfahren ist die chirurgische Behandlung durch die Lymphologische Liposculptur nach Cornely™.

**Ziel der Arbeit:**

Nach der extensiven Absaugung an den Extremitäten verbleibt eine ausgedehnte subkutane Wundhöhlenbildung mit einem trabekulären bindegewebigen Gerüst. Dennoch kommt es nur selten zu operationsbedingten Komplikationen. Das postoperative Management und die Gabe von Antibiotika und Antithrombotika wurden überprüft. Die Therapien der Komplikationen werden im Einzelnen dargestellt.

**Material und Methode:**

Retrospektiv wurde die Häufigkeit unerwünschter Nebenwirkungen bei 1400 Operationen von LiDo im Zeitraum 2020 ausgewertet. Das mittlere Alter der Patientinnen war 47,81 Jahre („range“ 16 bis 78 Jahre). Es wurden 504 Beine bds. außen (äußere Hälfte der Extremität [BA]), 504 Beine bds. innen (innere Hälfte der Extremität [BI]) und 392 Arme [A] bds. operiert.

**Ergebnisse:**

Relevante Nebenwirkungen traten als Infektionen (1,79 %), Serome (0,79 %), Erysipele (0,28 %), Nekrosen (0,14 %) und tiefe Beinvenenthrombosen (0,07) selten auf.

**Diskussion:**

Wir konnten in dem Regime der Lymphologischen Liposculptur™ zur operativen Behandlung der LiDo die Rate der postoperativen Komplikationen auf 3,07 % senken. In ihrer Metaanalyse zur Liposuktion berichten Kanapathy et al. über einer Gesamtinzidenz größerer chirurgischer Komplikationen von 3,35 %. Die Gesamtinzidenz leichtgradiger chirurgischer Komplikationen betrage 11,62 %, wobei Serom (5,51 %) die häufigste kleinere Komplikation gewesen sei [26]. Kruppa et al. berichten, dass das Liposuktionsverfahren einschließlich auch der aus ästhetischer Indikation durchgeführten Fettentfernungen mit einer Komplikationsquote von 9,5 % belastet sei. Hierbei stehen die Wundinfektionen mit 4,5 % und die Erysipelbildung mit 4 % deutlich im Vordergrund [20].

## Background

Lipohyperplasia dolorosa (LiDo), formerly called “lipedema,” is a chronic and—in terms of pressure pain—progressive disease that occurs almost exclusively in women and is characterized by a fat tissue distribution disorder with marked disproportion between the trunk and the extremities [1, 7, 9, 11, 12, 18]. The clinical presentation is highly multifaceted. Despite increasing research activity, there are still only a few objectifiable findings and reliable insights into the pathophysiology of the disease [2, 16, 17, 20].

Magnetic resonance lymphangiography (MRL) usually confirms an orthologous lymphatic vasculature. However, quantitative measurements of indirect functional lymph scintigraphy of lymph flow could demonstrate an age-correlated high-volume transport insufficiency [4–7, 21, 22, 24]. In the context of orthologous lymphatic anatomy, this functional insufficiency is to be evaluated as pathological and was named the “Marsch thesis” by Cornely after its describer [11, 14, 21]. It is a known pathophysiological lymphological finding underlying the symmetrical pressure-painful adipose tissue distribution disorder in LiDo. However, the diagnosis of this lymphological clinical picture follows at first sight the clinical appearance.

In addition to conservative treatment with complex decongestive therapy (CDT) with manual lymphatic drainage (MLD) and compression, surgical treatment of LiDo, in this case as two-part Lymphological liposculpture, i.e., surgery under tumescent local anesthesia (TLA) and postoperative accentuated manual lymphatic drainage (AMLD), has become established [10, 11, 13, 14].

There are three goals of intervention for Lipohyperplasia dolorosa:

- improvement and ideally complete elimination of pressure pain,
- avoidance of further CDT [15],
- harmonization of the volume ratio between the extremities and trunk.

**Fig. 1:**
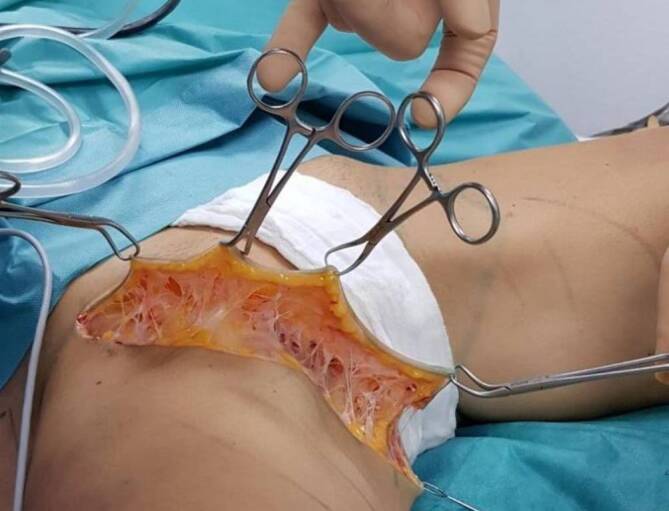
Exemplary illustration of surface enlargements due to the formation of channels and cavities using the example of an empty, suctioned “degreased” tissue prepared for flap plastic surgery according to Avelar. The connective tissue framework with residual fatty tissue on the skin is clearly visible. Such insights are of course not possible with the Lymphological Liposculpture resection performed as suction in Lipohyperplasia dolorosa

During the surgical procedure, removal of the subcutaneous fatty tissue creates a wound cavity with a considerable increase in surface area, which corresponds to 18% of the body surface ([23]; Fig. 1). The formation of edema, which can be the starting point of infections and other postoperative complications, is possible.

## Materials and methods

The files of the 504 LiDo patients operated on in 2020 were systematically evaluated for complications directly related to the operation and their treatment. A total of 140 Lymphological Liposculptures had been performed on the patients with LiDo. The mean age of these patients was 47.81 years (range 16–78 years); 504 external legs (outer half of the limb [BO]), 504 internal legs (inner half of the limb [BI]), and 392 external arms [A] had been operated.

In this research with patients’ personal data, the Declaration of Helsinki in its current version was observed. According to the North Rhine-Westphalian Data Protection Act (Cologne), retrospective analysis and anonymized reporting of patient data without informed consent is appropriate and ethics approval is not required.

### Lymphological liposculpture

As a rule, three procedures at intervals of no less than 4 weeks are required to achieve circular suction of the extremities [27].

The preferred order is

1. operation on the outer 50% of both legs (BO),
2. operation of the two arms (A),
3. surgery of the inner 50% of both legs (BI).

The TLA is inserted under simultaneous analgesia or general anesthesia. A motor-driven suction system is used to support the suction (power-assisted liposuction, PAL). Finally, the incisions in the skin are not closed to ensure open drainage of the wound secretion. The postoperative phase is dedicated to rebalancing lymph transport and improving high-volume transport insufficiency. For this purpose, modified CDT is performed as accentuated manual lymphatic drainage (AMLD) with compression and physical treatment for 4 weeks at a time. This AMLD includes 24‑h compression of the operated extremities with textile knitted fabric for 7 days with subsequent reduction to 12 h during the day or at night at the patient’s discretion as well as 10 manual lymph drainages as a whole therapy, accentuated on the operated extremity, starting with four AMLD per week and then weekly reduction to one AMLD per week.

For perioperative infection prophylaxis, after intraoperative single-shot antibiosis with 2 g of cephazolin i.v., cefuroxime 500 mg is given orally in a weight-adapted scheme for the first 5 days after surgery. In case of cefuroxime allergy, clindamycin is available in the standard dosage. Likewise, 3‑day thrombosis prophylaxis with low-molecular-weight enoxaparin sodium 40 mg (Clexane® 4000 I. E.) is standard for Lymphological Liposculpture.

## Results

In 2020, 1400 operations were performed for LiDo; 504 BO, 504 BI, and 392 A.

After 24 of the 504 BO (4.76%), 10 inflammations, 9 seromas, 2 erysipelas, 2 necroses, and 1 DVT were treated. In 16 of the 504 BI (3.17%), 13 inflammations, 2 seromas, and 1 erysipelas occurred. In 3 of the 392 (0.76%) operations on arms, 2 inflammations and 1 erysipelas had to be treated postoperatively.

Accordingly, a total of 43 (3.07%) of these 1400 Lymphological Liposculpture procedures in LiDo patients were accompanied by adverse side effects as a complication of surgery within 30 days (Table 1).

**Table 1:** Adverse effects after Lymphological Liposculpture (*n* = 1400 for the observation period 2020)

Complication	Percentage	Absolute value for 2020 (*n* = 1400)
Inflammation	1.79	25
Seroma	0.79	11
Erysipelas	0.28	4
Necrosis	0.14	2
Thrombosis	0.07	1
Σ	3.07	43

Infection occurred in 25 patients (1.79%) and seroma formation in 11 patients (0.79%). Erysipelas developed in 4 patients (0.28%), dry necrosis in 2 patients (0.14%), and deep vein thrombosis in 1 patient (0.07%).

## Discussion

### Complications and their management

In a large multicenter American study on safety and complications after liposuction in TLA, an overall rate of 1.12% was published by Hanke in 1995 [19]. The Safety of Tumescent Liposuction in 15,336 Patients study examined the results of 66 dermatosurgeons who contoured a total of approximately 44,000 body areas. Complications included 0.38% scrotal and labial edemas or ecchymosis, 0.33% infection, 0.26% permanent skin irregularity, 0.09% persistent postoperative edema, 0.05% excessive or persistent postoperative pain, and 0.01% unacceptable scarring. The low complication rate may be due to the consistently cosmetic indication of liposuction performed exclusively by dermatologists. The aspirate quantities were smaller compared to the medical indication in LiDo, and the postoperative subcutaneous surface expansion was therefore lower.

In their 2019 review, Halk et al. re-emphasized the safety of liposuction using tumescent local anesthesia (TLA), especially when no or minimal systemic anesthesia is used. Performance of this technique has been shown to be safe in practice. Knowledge and training on how to perform the tumescent procedure is crucial to ensure optimal safety [25].

In 2020, Kruppa et al. stated a complication rate of approximately 9.5% for all—both cosmetically and medically—indicated liposuctions in their review paper [20]. Bleeding complications account for 1%, wound infections for 4.5%, and development of erysipelas for a further 4%.

Kanapathy M et al. reported on 3583 patients in their meta-analysis in 2021. The overall incidence of major surgical complications was 3.35%. The most common major complication was blood loss requiring transfusion (2.89%), followed by pulmonary embolism (0.18%), hematoma (0.16%), necrotizing fasciitis (0.13%), and deep vein thrombosis (0.12%). No fat embolisms or deaths were reported in the included studies. The overall incidence of minor surgical complications was 11.62%, with seroma being the most common minor complication (5.51%) [26].

Among the 1400 operations in the 504 patients with LiDo, a complication rate of 3.07% was determined in 2020. BO were more frequently affected (4.76%) than BI (3.17%). It is noticeable that complications on the arms are unusually rare, at 0.76%. No patient had had previous procedures on the extremities, and no patient had more than one complication. There was no preference for one side of the body. A correlation of patient age and surgery-related adverse events was also not found. All complications healed under the specific outpatient treatment. There were no deaths and no cases of necrotizing fasciitis.

The low rate of complications may also be due to the obligatory postoperative program over 4 weeks (AMLD), which is part of the Lymphological Liposculpture surgical procedure. Fluid retention occurs most frequently in the lower legs, less frequently in the thighs, and rarely in the arms. Such secretion stasis may be conducive to infection, because on the legs, stasis-related adverse effects seem to occur more on the lower legs than on the upper legs. There is obviously a stasis-related risk of infection that can be reduced by AMLD.

Perioperative antibiotic administration plays an equally important role in the prevention of postoperative wound infection to that of AMLD. Intraoperative single-shot antibiotics with 2 g of cephazolin i.v. are followed by postoperative administration of cefuroxime orally (weight adjusted; 2 × 250 mg cefuroxime up to 70 kg body weight, 2 × 500 mg cefuroxime above 70 kg body weight) for 5 days to minimize this risk.

The treatment corresponding to the clinical presentation was carried out as follows.

### Inflammation

After the operation, open drainage is aimed for. The incision sites are covered with fluid-absorbing compresses.

**Fig. 2:**
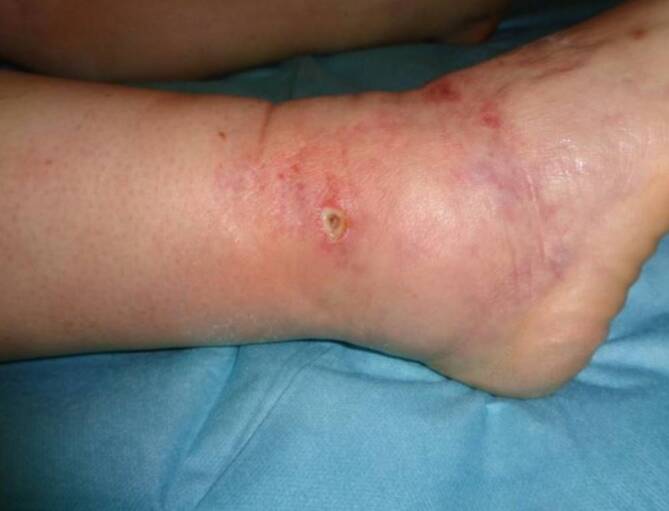
Redness with swelling in the area of the incision, incipient stitch canal infection

Localized redness or overheating with edema in the area of the incision (Fig. 2) does not require systemic therapy. Cooling with cold black tea compresses several times a day is the best option from a dermatological point of view because of its astringent effect. Antiseptic compresses are not necessary. However, if the localized redness remains without a tendency to regress or if exacerbation occurs, treatment with a topical antibiotic, e.g., Fucicort® (20 mg fusidic acid and 1 mg betamethasone/1 g ointment) should be used, taking into account allergological local reactions. Routine systemic postoperative antibiotics are continued with cefuroxime in case of infection until the antibiogram is available and then adjusted if necessary. AMLD promotes de-edema and wound healing, and muscle activation through exercise is essential for decongestion. Bed rest is not indicated.

### Abscess

The most common cause is secondary bacterial colonization of a seroma or hematoma. Treatment is systemic after antibiogram. Sometimes surgical draining measures are necessary. However, extensive surgical interventions, such as vacuum sealants, are not indicated after surgical removal of the subcutaneous tissue and the resulting tissue cavity. Drainage of abscess fluid through flaps is preferable. Silicone drains (e.g., Silastic©, Dow Corning, Midland, MI, USA) allow sufficient secretion drainage.

### Seroma

**Fig. 3:**
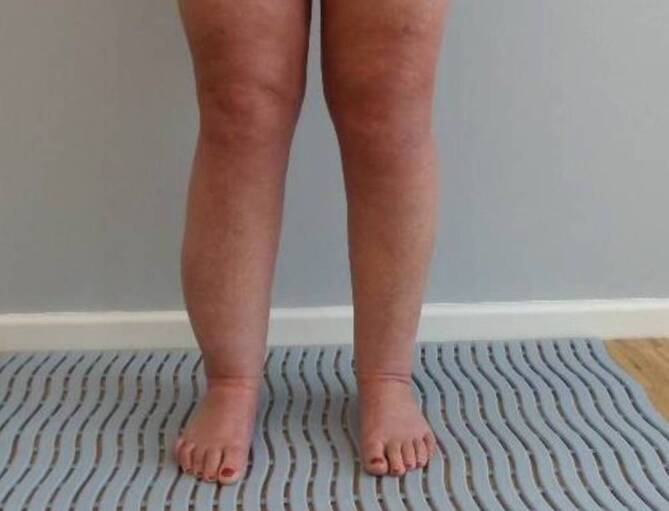
Seroma formation on the right lower leg

Clinically, a locally circumscribed fluctuating swelling can be palpated with an inconspicuous skin–soft tissue mantle and largely painless manifestation (Fig. 3).

In the case of smaller seromas, relieving surgical intervention is not necessary. An eccentric compression with increased pressure usually leads to sufficient resorption of the secretion. Sometimes sterile puncture of the seroma may be necessary due to pronounced fluctuation and frequent pressure pain. Eccentric compression is mandatory after seroma relief by this means. If the initial measure is not sufficient, further punctures may be performed.

### Erysipelas

**Fig. 4:**
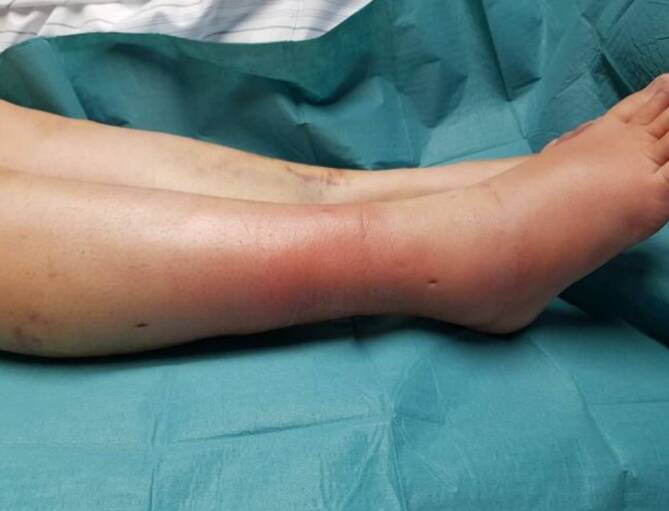
Postoperative edema, swelling, and sharply demarcated hyperthermic erythema as a local clinical sign of erysipelas. Systemically, there is obligatorily a marked increase in body temperature

Clinically, the triad of hyperthermic, sharply demarcated erythema, fever, and lymphadenitis appears (Fig. 4). This infection is caused by beta-hemolytic group A streptococci, more rarely by *Staphylococcus aureus *[8]. The diagnosis requires the obligatory symptom of fever. Typical of erysipelas is a rapidly spreading bright red erythema, sharply demarcated at the front and painful. Symptoms can range from small punctate hemorrhages (petechiae) without accompanying symptoms to a highly febrile infection with chills and severe impairment of the general condition. In some cases, bullae and even hemorrhagic bullae may form.

Skin swabs are not a basis for antibiotic treatment of erysipelas. A pretherapeutic blood culture is necessary. In contrast to the usual treatment of erysipelas with penicillin, postoperative erysipelas in LiDo must be treated with a broad-spectrum antibiotic, preferably a cephalosporin preparation, due to the germ spectrum. A 5-day antibiotic treatment with cefuroxime 500 mg twice daily is therefore standard in Lymphological Liposculpture, also for erysipelas, until arrival of the antibiogram after the blood culture. In case of allergy to cephalosporins, weight-adjusted sultamicillin (e.g., Unacid PD oral®, Pfizer, New York, NY, USA), in case of suspected cross-allergy, clindamycin or ciprofloxacin, can be used in typical dosage. If treatment fails prematurely, further antibiotics should be administered only after an antibiogram and, if necessary, after a new blood culture. Erysipelas must be treated with oral or intravenous antibiotics for 8–10 days. Systemic antiphlogistic therapy, e.g., with ibuprofen up to 2.4 g/d, is obligatory.

Cooling black tea compresses have an astringent effect and are indicated from a dermatological point of view, as are cooling e.g. Octenisept® (Schülke & Mayr, Norderstedt, Germany) compresses when used for a short time.

From the second day of the infection, manual lymphatic drainage should be resumed under antibiotic and antiphlogistic therapy with exclusion of the painful areas for decongestion. The spread of germs is not to be feared under antibiotic protection. Bed rest or immobilization is not indicated. Thrombosis prophylaxis can be dispensed with during mobilization.

### Necrosis

With progressive inflammatory reaction and bacterial colonization, necrosis may occur in very rare cases. This dry necrosis is usually the result of a local circulatory disorder. Further treatment is conservative. Dry covering, daily dressing changes, and waiting for demarcation of the necrotic zone are the main priorities. Daily showering followed by drying by careful dabbing to cleanse the wound is desirable. In dry necroses without signs of inflammation in the surrounding area, further antibiotic therapy can be dispensed with. Invasive surgical measures, especially necrectomy with vacuum sealing, should be avoided. Such interventions promote large defects in the pronounced shrinkage of the skin–soft tissue mantle desired postoperatively after LiDo surgery, which can rarely be closed primarily and often require skin grafting. Minimally invasive measures such as insertion of silicone drains (e.g., Silastic©) efficiently relieve the tissue. The drainage flap should not be pulled directly under the necrosis but should drain off the adjacent abscess formations.

### Thrombosis

Deep vein thrombosis is an absolute rarity after Lymphological Liposculpture of lipohyperplasia dolorosa.

If premature closure of the caudal incision at the ankle occurs, the wound secretion may collect there. Lack of mobility increases the pressure painful swelling in the area of the affected lower leg. The pressure pain is often misinterpreted by patients as leg vein thrombosis. An active venous pump ensures regulated blood flow, manual lymphatic drainage and compression relieve the local swelling.

In addition to compression stockings, early mobilization after surgery, sufficient fluids, and a short 3‑day drug treatment with low-molecular-weight enoxaparin sodium 40 mg (Clexane® [Sanofi Pasteur MSD, Paris, France] 4000 I. E), exercise seems to be the best thrombosis prophylaxis in Lymphological Liposculpture. In case of doubt, however, treatment should first be given according to guidelines, as in the case of a reliably proven thrombosis, and this diagnosis should be verified by further imaging examinations and clinical controls [3].

#### Limitations of the study.

A possible correlation between complications and the amount of aspirate was not investigated. We will take this into account in the next retrospective data analysis on the safety of the surgical procedure as well as the subclassification according to LiDo stages and a differentiation of the occurrence of complications on the thigh and lower leg. Clinically, the lower legs are the first localization of leg complications, but unfortunately, these data were not separated.

## Conclusion

Resection of the fatty tissue of Lipohyperplasia dolorosa (“lipedema”) is often mistakenly equated with an esthetically motivated operation and attributed to liposuction performed for cosmetic indications.

LiDo can extend from the wrist to the shoulder or from the ankle to the groin. For the correct treatment of this clinical picture, subtotal removal of this subcutaneous fat tissue between fascia and dermis is necessary. During the operation, extensive large wound areas are created on the extremities. Postoperative treatment with manual lymphatic drainage and compression as AMLD is a tried-and-tested procedure to counteract secretion stasis that promotes complications.

Postoperative management of Lipohyperplasia dolorosa includes

- immediate full mobilization,
- AMLD with compression and physical treatment for 4 weeks each,
- 5 days of antibiotic treatment with cefuroxime 500 mg twice daily (in case of allergy, clindamycin 600 mg or ciprofloxacin 500 mg twice daily and weight-adapted sultamicillin 375 [Unacid PD oral®] twice daily are available as alternatives), and
- 3 days of s.c. thrombosis prophylaxis with low-molecular-weight enoxaparin sodium 40 mg (Clexane® 4000 I. E) as standard procedure.

We were able to reduce the rate of postoperative complications to 3.07% in this regime of Lymphological Liposculpture for surgical treatment of Lipohyperplasia dolorosa.

With the current work, we confirm the safety and good tolerability of the presented procedure for LiDo on the arms and legs.

## Declarations

For this article no studies with human participants or animals were performed by any of the authors. All studies mentioned were in accordance with the ethical standards indicated in each case.

The supplement containing this article is not sponsored by industry.
